# Virtual reality enhances affective valence and attentional dissociation during exercise but not exercise-induced hypoalgesia

**DOI:** 10.1007/s10055-026-01336-z

**Published:** 2026-04-13

**Authors:** Jonathan M. Bird, Pedro F. Lima, Jasmine Le Grice, Sam W. Hughes

**Affiliations:** 1 Department of Targeted Intervention, University College London, London, UK; 2 Department of Clinical and Biomedical Sciences, University of Exeter, Exeter, UK

**Keywords:** Endogenous analgesia, Extended reality, Immersion, Pain management, Physical activity

## Abstract

The analgesic potential of virtual reality has been demonstrated in acute and chronic pain populations, where it is often employed as a distraction tool or integrated into virtually delivered biopsychosocial pain management plans. However, the extent to which virtual reality can augment exercise-induced hypoalgesia remains underexplored. The aim of the study was to examine the effects of virtual reality on experimentally-induced pain (using cuff pressure algometry), as well as affective and perceptual responses, to moderate-intensity cycle ergometer exercise. A counterbalanced within-subjects design was employed and 19 healthy, pain-free adults completed 20-min exercise bouts at ventilatory threshold under two conditions: virtual reality and control. A one-way repeated measures ANOVA indicated a significant main effect of assessment for pressure detection threshold (*p* = .038), with higher pain thresholds observed immediately following each exercise bout when compared to baseline. However, there were no differences in pressure detection threshold between exercise conditions. Analyses also revealed that the virtual reality condition was associated with more positive affective valence (*p* = .002) and greater attentional dissociation (*p* = .036) when compared to control, as well as higher post-task ratings of exercise enjoyment (*p* < .001), remembered pleasure (*p* = .045), and forecasted pleasure (*p* = .033). Despite the lack of enhancement in experimentally-induced pain modulation, the findings support the notion that virtual reality technology can be a useful tool for promoting pleasurable exercise experiences.

## Introduction

Physical inactivity is increasing globally. The upshot of this is a surge of obesity, type 2 diabetes, and cardiovascular disease (Santos et al. 2023). Unfortunately, recent estimates show that nearly one third of adults globally (1.8b) are insufficiently active and that, if current trends continue, the World Health Organization’s global target to reduce physical inactivity by 15% by 2030 will remain unmet (Strain et al. 2024). Exercise, a sub-category of physical activity that is planned, structured and repetitive, can improve both physical and mental health (Isath et al. 2023; Solmi et al. 2025). Despite its benefits, adherence to exercise programmes remains particularly challenging.

Psychological hedonism holds that individuals are likely to perform activities in which they derive pleasure and avoid activities in which they derive displeasure (Kahneman 1999). Indeed, there is evidence to suggest that such feelings states are indicative of future exercise behaviour (Teixeira et al. 2024). Hence, interventions are needed to create pleasurable experiences that could conceivably enhance exercise adherence. Virtual reality (VR) is a promising technology in this regard and can be used to deliver interactive simulations that have the potential to prompt high perceptions of presence (i.e., the sense of being transported to another environment; Slater 2018).

### Virtual reality exercise

There is a growing corpus of work supporting the use of VR during exercise (Barbour et al. 2024; Bruce et al. 2025; Bird et al. in press). For example, a registered report by Bird et al. (2024) involved an examination of four technologies (i.e., television, augmented reality, 360° video, VR) during cycle ergometer exercise performed at ventilatory threshold (VT). The findings indicated that the VR condition prompted the most positive in-task affective valence (i.e., feelings of pleasure vs. displeasure) and greatest attentional dissociation. Moreover, the VR condition elicited the highest scores for enjoyment, remembered pleasure, and forecasted pleasure, all of which were measured upon the cessation of each trial. Despite these encouraging findings, the mechanisms underlying the positive effects of VR-assisted exercise remain unclear.

### Exercise-induced hypoalgesia

Participation in a single bout of exercise typically leads to an acute reduction in pain sensitivity among healthy, pain-free adults, and is often referred to as *exercise-induced hypoalgesia* (EIH; Rice et al. 2019; Vaegter and Jones 2020). This is typically assessed by examining pain thresholds (i.e., the minimum intensity of noxious stimulus perceived as painful; Song et al. 2023) pre- and post-exercise. Hypoalgesia has been reported in response to several exercise modalities including aerobic (Niwa et al. 2022), isometric (Wu et al. 2022), and dynamic resistance (Lyons et al. 2025).

The analgesic potential of VR has also been demonstrated in acute and chronic pain populations, where it is often employed as a distraction tool to divert attention from pain (Moreau et al. 2024) or integrated into virtually delivered biopsychosocial pain management plans (Medina et al. 2024). However, the role of VR during active physical engagement, such as exercise, is less clear. EIH has been attributed to the activation of endogenous analgesic systems (Koltyn 2000; Naugle et al. 2012; Koltyn et al. 2014; Bannister and Hughes 2023). While VR may feasibly amplify these analgesic processes by enhancing attentional engagement or endogenous analgesic mechanisms (Smith et al. 2025), its capacity to augment EIH remains underexplored. It is plausible that experimentally-induced pain responses may help explain the positive affective and perceptual outcomes observed in the extant VR-exercise literature (Bird et al. 2024; Barbour et al. 2024).

### Aims and hypotheses

The aim of the study was to examine the effects of VR on experimentally-induced pain (using cuff pressure algometry), as well as affective and perceptual responses, to moderate-intensity exercise performed at VT. This exercise intensity was chosen as it falls within a zone that is associated with a high degree of inter-individual variability in affective valance (Ekkekakis 2003). Cuff pressure algometry was employed to help determine changes in pressure pain detection thresholds (PDT) and endogenous analgesia, assessed via conditioned pain modulation (CPM; Graven-Nielsen et al. 2015). We sought to determine whether VR could amplify EIH among a healthy, pain-free population. To that end, the study entailed exercising under a VR condition and a control. We hypothesised that VR would enhance exercise-induced effects on PDT (*H*_1_) and conditioned PDT (*H*_2_). Moreover, we predicted that VR would prompt more positive affective valence (*H*_3_), higher affective arousal (*H*_4_), reduced ratings of perceived exertion (RPE; *H*_5_) and greater attentional dissociation (*H*_6_) when compared to control. We also predicted that VR would be associated with greater post-exercise scores pertaining to perceived enjoyment, remembered pleasure, and forecasted pleasure, when compared to control (*H*_7_).

## Method

### Registration

The study was preregistered and all data are publicly available via the Open Science Framework (https://doi.org/10.17605/OSF.IO/BU85T). We preregistered the hypotheses, study protocol (including design plan and sampling plan), and analysis plan. The original preregistration incorporated planned analyses for pressure pain tolerance (PPT) and temporal summation of pain (TSP) data. However, early testing revealed inconsistencies regarding whether participants reached cut-off, rendering analyses of these data unsuitable. This is something that should be taken into consideration in the design of future pain-related research studies. Accordingly, we removed these analyses and their associated hypotheses. Furthermore, we replaced our initial power analysis, which was predicated on PPT data, with a more suitable alternative.

### Participants

Two power analyses were conducted in RStudio (v. 2023.12.1) to inform the sample size. First, the pwr package (Champely et al. 2020) was used with power of 0.80, alpha of 0.05 and *f* of 0.55 in relation to the effects of VR technology on PDT (Tesarz et al. 2024). The analysis indicated that 12 participants would be required. Second, the Superpower package was used for affective variables in accordance with guidance for factorial analyses (Lakens and Caldwell 2021). Specifically, the extant literature helped inform the predicted pattern of means for affective valence between two conditions (i.e., VR and control) and four timepoints (Bird et al. 2019, 2021). A power curve indicated that 14 participants would be required to obtain 80% power in relation to a 2 (Condition) × 4 (Timepoint) interaction.

The study was approved by the University of Exeter Research Ethics Committee (Ref: 3330643). Inclusion criteria ensured that participants were aged between 18 and 30 years, safe to engage in moderate-to-vigorous physical activity according to the Physical Activity Readiness Questionnaire (PAR-Q; Warburton et al. 2011), without any visual impairment and/or hearing deficiency and familiar with cycle ergometry. Potential participants were also pre-screened for susceptibility to motion sickness using the Visually Induced Motion Sickness Susceptibility Questionnaire (VIMSSQ; Golding et al. 2021). Participants were excluded from the investigation if they indicated avoidance to using head-mounted displays. Nineteen healthy, pain-free adults were recruited to take part in the present investigation (see Table 1).

**Table 1:** Participant characteristics

Characteristic	*n*	%	M	SD
Gender				
Female	10	52.63		
Male	9	47.37		
Nationality				
British	17	89.47		
French	2	10.53		
Ethnicity				
Asian or Asian British	1	5.26		
Mixed or multiple ethnic groups	2	10.52		
White	16	84.21		
Age (years)			22.42	1.50
Height (m)			1.73	0.10
Weight (kg)			66.58	11.07
BMI (kg/m^2^)			22.03	2.17

BMI = body mass index

### Experimental procedures

Participants were required to attend a laboratory on two occasions (i.e., one habituation/incremental exercise session and one experimental session). During the initial visit, volunteers confirmed their eligibility to take part in the study by completing the PAR-Q (Warburton et al. 2011), VIMSSQ (Golding et al. 2021), and a demographic questionnaire. Participants were provided with an information sheet and afforded the opportunity to ask questions about the investigation. Subsequently, each participant provided informed consent. Participants were habituated to each of the measures and items of equipment (i.e., cycle ergometer, VR headset and cuff pressure algometry) that were to be employed during the experimental session. Thereafter, each participant was required to complete an incremental exercise test on an electronically braked cycle ergometer in order to determine their VT (see Bird et al. 2024 for full details).

**Fig. 1:**
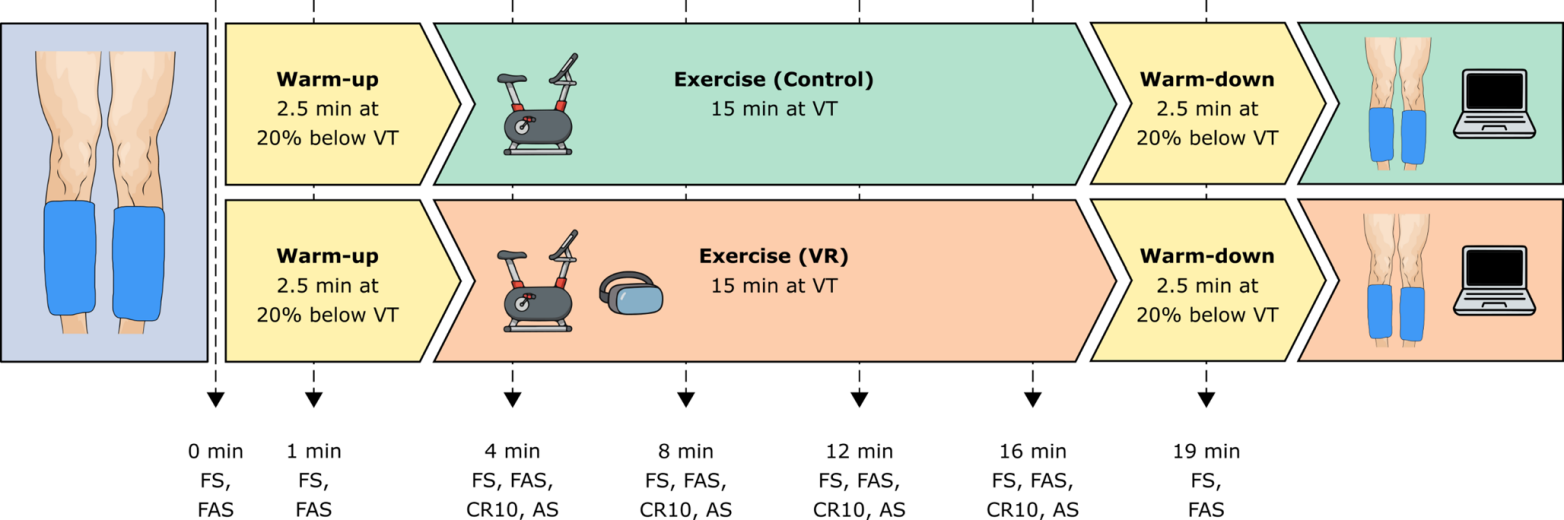
Overview of experimental testing. VR = virtual reality; VT = ventilatory threshold; FS = Feeling Scale; FAS = Felt Arousal Scale; CR10 = Borg CR10 Scale; AS = Attentional Scale.

A counterbalanced within-subjects design was employed and experimental testing took place at least 24 h after the initial habituation session. The experimental testing entailed exercising under two conditions (i.e., VR and control) separated by a 15 min period of seated rest. Each trial involved a 2.5 min warm-up at 20% below VT, 15 min exercise at VT and a 2.5 min warm-down at 20% below VT (see Fig. 1). Participants were required to maintain a cadence of 75 ± 3 rpm throughout each trial. Each participant could view their cadence directly on the cycle ergometer in the control condition. The second and third authors monitored participants’ cadence in the VR condition and provided verbal feedback where necessary (Bird et al. 2021).

**Fig. 2:**
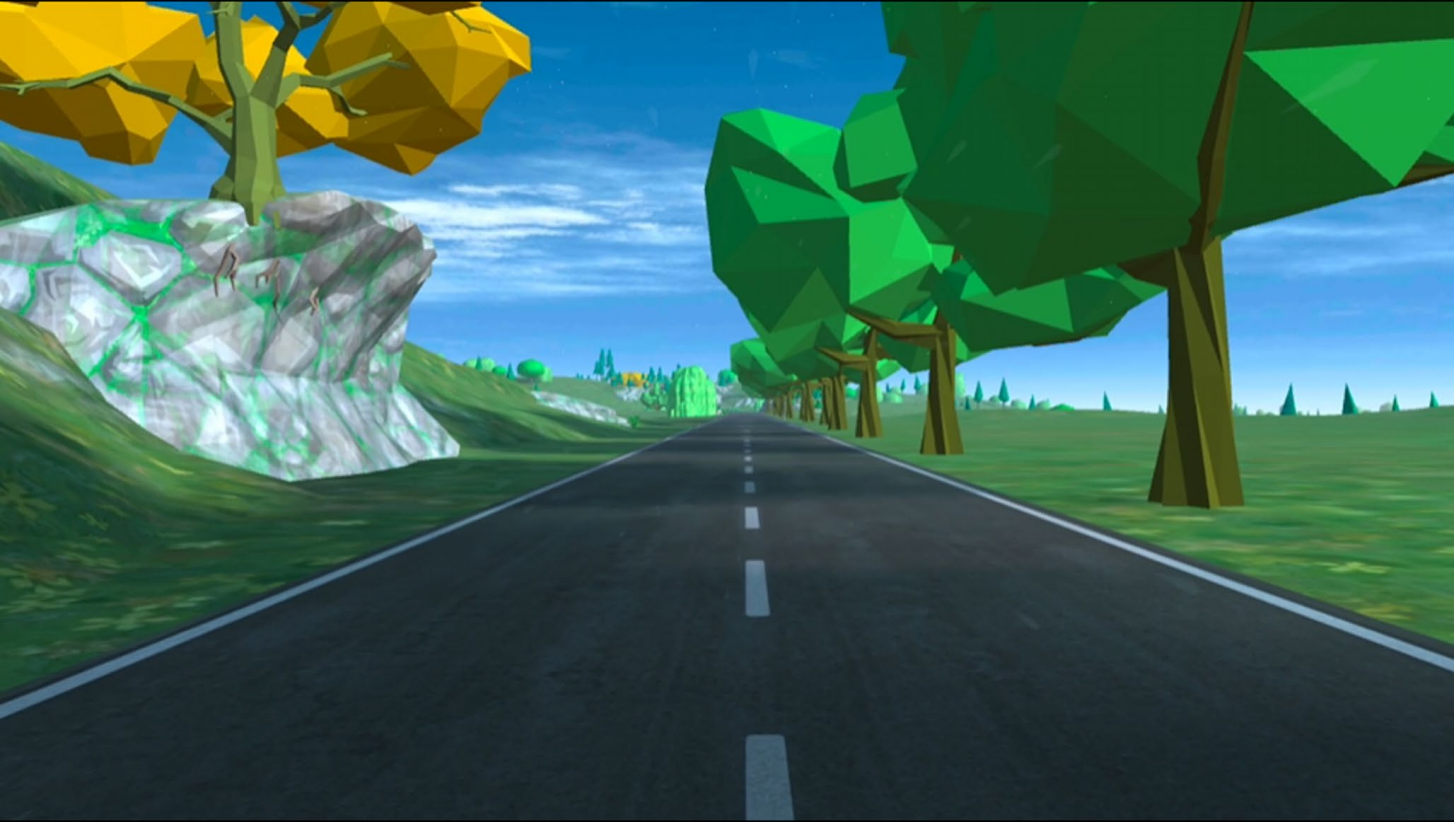
Visual stimuli used in the virtual reality condition. Visual stimuli courtesy of VirZOOM (Cambridge, MA, USA).

The *VZfit* application (VirZOOM, Cambridge, MA) was used on a Quest 2 headset (Meta, Menlo Park, CA) in the VR condition. This application allows users to cycle through a virtual countryside scene from a first person perspective (see Fig. 2) and was chosen for its high degree of congruence to the exercise task in physical reality. The visuals were responsive to changes in pedal cadence and the participant was able to steer the virtual bike in real-time by tilting their head (i.e., in the roll plane). Participants did not require the use of hand controllers during the exercise bout. Multiplayer capabilities were turned off, as was music, with the only audio consisting of environmental sound (e.g., birdsong, tyre-road interaction).

### Measures

Pain thresholds and CPM protocols were conducted with a computer-controlled cuff-algometry system (LabBench software 4.7.3 and CPAR+ device, Aalborg, Denmark). Participants sat on a padded chair with leg support raised to approximately 45 degrees. Independent computerised torniquet cuffs were placed around each calf (10 × 61 cm). Test and conditioning stimuli were always applied to the dominant and non-dominant legs, respectively. Participants were handed a computerised visual analogue scale (VAS) anchored from ‘no pain’ to ‘maximal pain’ and a slider set to ‘no pain’ as a default. PDT (dominant leg) was determined as the pressure at which the test stimulus was first perceived as painful. Upon reaching PDT, each participant was required to continuously rate their pain intensity during further cuff inflation. Subsequently, pressure pain tolerance (PPT) was defined as the point at which the pain was no longer tolerable. Following a 2 min resting period, the final part of the CPM protocol was carried out. The conditioning stimulus cuff (left calf) inflated rapidly to a pressure equal to 70% participants’ PTT on the same calf, and it remained inflated until the end of the protocol to serve as a concurrent conditioning tonic stimulus. Once it reached the target pressure, the test stimulus cuff started to inflate at a rate of 1 kPa/sec. Participants were instructed to rate the test stimulus (right calf) in the exact same way as they did during thresholding. All participants underwent this cuff algometry protocol at baseline and then immediately following the cessation of VR and control conditions (see Fig. 1). The CPM effect in each condition was calculated as the difference between the conditioned PDT and PDT responses.

Core affect was assessed at 0, 1, 4, 8, 12, 16 and 19 min of each exercise bout to capture the dynamic nature of exercise-related affect (Bird et al. 2019). However, only data obtained during the main exercise phase at VT (i.e., 4, 8, 12, and 16 min) were subject to statistical analysis in relation to *H*_3–4_. Affective valence was measured using the Feeling Scale (FS; Hardy and Rejeski 1989), which is a single-item, 11-point scale anchored by -5 ([I feel] *very bad*) and 5 (*very good*). Arousal was assessed using the Felt Arousal Scale (FAS; Svebak and Murgatroyd 1985), which is a single-item, six-point scale anchored by 1 (*low arousal*) and 6 (*high arousal*). Perceptual variables were measured during the main exercise phase, after 4, 8, 12 and 16 min. RPE was measured using the Borg CR10 Scale (CR10; Borg 1998), which is anchored by 0 (*nothing at all*) and 10 (*extremely strong*). State attentional focus was assessed using the Attentional Scale (AS; Tammen 1996), which is anchored by 0 (*internal focus* [bodily sensations, heart rate, breathing, etc.]) and 100 (*external focus* [daydreaming, external environment, etc.]). Participants were required to respond to these measures verbally and could see them during the control condition. During the VR condition, the second and third authors communicated the anchors of each measure at the time of response to facilitate recall.

Upon completion of each trial, exercise enjoyment was measured using the Physical Activity Enjoyment Scale (PACES; Kendzierski and DeCarlo 1991), which is comprised of 18 items attached to 7-point scales (e.g., 1 = *I enjoy it*, 7 = *I hate it* [item 1]). Remembered pleasure was assessed by means of a visual analogue scale that accompanied the question “How did the exercise session make you feel?” The scale ranges from − 100 (*very unpleasant*) to 100 (*very pleasant*) in intervals of 1 (Zenko et al. 2016). Forecasted pleasure was measured using the Empirical Valence Scale (Lishner et al. 2008). Each participant responded to the question “If you repeated the exercise session again, how do you think it would make you feel?”. Participants were required to select one of fifteen descriptors depicted underneath the scale, ranging from − 100 (*most unpleasant imaginable*) to 100 (*most pleasant imaginable*). As a manipulation check, perceptions of presence were assessed in relation to the VR condition using the Spatial Presence Experience Scale (SPES; Hartmann et al. 2016). The SPES comprises eight items attached to a 5-point scale anchored by 1 (*I do not agree at all*) and 5 (*I fully agree*).

### Data analysis

The analysis plan was registered via the Open Science Framework and all analyses were conducted in RStudio (v. 2024.09.0). Robust *z* scores (± 3.29), predicated on medians and median absolute deviations, were calculated using the performance package (Lüdecke et al. 2021) to identify the presence of univariate outliers. Two participants were subsequently removed from the dataset, as their removal remedied the majority of outliers across dependent variables (i.e., 7 out of 12; 58.33%). Remaining outliers were treated by adjusting the raw score by one unit smaller or larger than the next most extreme score in the distribution until *z*_robust_ < ± 3.29 (Tabachnick and Fidell 2019). Checks were made for the parametric assumptions underlying repeated-measures (RM) ANOVA. For example, the distributional properties of the data were examined visually by means of Q–Q plots and histograms (Coolican 2018). We found minor violations of normality in 14 cells of the analysis (10 at *p* < .05, four at *p* < .01). However, we did not transform these data given concerns regarding the transformation of data derived from Likert scales (see e.g., Nevill and Lane 2007).

Pain responses (i.e., PDT and conditioned PDT) were analysed by means of one-way RM ANOVAs. Affective (i.e., affective valence and arousal) and perceptual (i.e., RPE and attentional focus) data, obtained during the main exercise phase at VT, were assessed using factorial 2 (Condition) × 4 (Timepoint) RM ANOVAs. Exercise enjoyment, remembered pleasure and forecasted pleasure were analysed by paired samples *t*-tests. Greenhouse–Geisser adjustments were made to *F* tests in which the sphericity assumption was violated. Data and analysis scripts can be located online (see 10.17605/OSF.IO/BU85T).

## Results

Descriptive statistics for all variables are presented in Tables 2 and 3.

**Table 2:** Descriptive statistics for pain variables

Variable	Baseline		Control		Virtual Reality	
	M	SD	M	SD	M	SD
PDT	27.17	10.56	32.68	13.08	35.92	17.90
PDT (conditioned)	35.97	11.16	37.97	14.21	38.95	15.79
∆PDT (CPM effect)	− 7.42	8.94	− 7.12	8.89	− 3.02	6.08

Statistics are presented following data cleaning processes. PDT = pressure detection threshold; CPM = conditioned pain modulation

**Table 3:** Descriptive statistics for affective, perceptual, and post-task variables

Variable	Control		Virtual Reality	
	M	SD	M	SD
Affective valence				
0 min	3.35	1.58	3.76	1.48
1 min	3.18	1.51	3.88	1.22
4 min	3.12	1.45	3.94	1.03
8 min	2.88	1.69	3.82	0.95
12 min	2.88	1.62	3.88	1.11
16 min	2.47	1.91	3.47	1.23
19 min	2.53	1.87	3.53	1.23
Arousal				
0 min	3.76	1.56	4.24	1.68
1 min	3.71	1.45	4.18	1.59
4 min	3.76	1.48	4.35	1.27
8 min	3.41	1.54	4.12	1.11
12 min	3.65	1.62	4.12	1.05
16 min	3.41	1.54	3.76	1.30
19 min	3.47	1.66	4.12	1.17
Perceived exertion				
4 min	4.62	2.00	4.53	1.81
8 min	5.26	2.03	4.88	1.73
12 min	5.76	2.28	5.65	1.66
16 min	6.18	2.43	6.18	1.74
State attention				
4 min	50.59	17.93	67.06	22.85
8 min	48.53	17.30	65.59	21.20
12 min	46.18	20.27	61.47	22.76
16 min	42.06	19.29	60.00	23.65
Exercise enjoyment	83.29	17.51	99.76	16.05
Remembered pleasure	22.76	47.69	42.65	32.28
Forecasted pleasure	12.88	33.94	34.35	34.82
Presence	–	–	29.94	5.08

Statistics are presented following data cleaning processes

### Pain responses

**Fig. 3:**
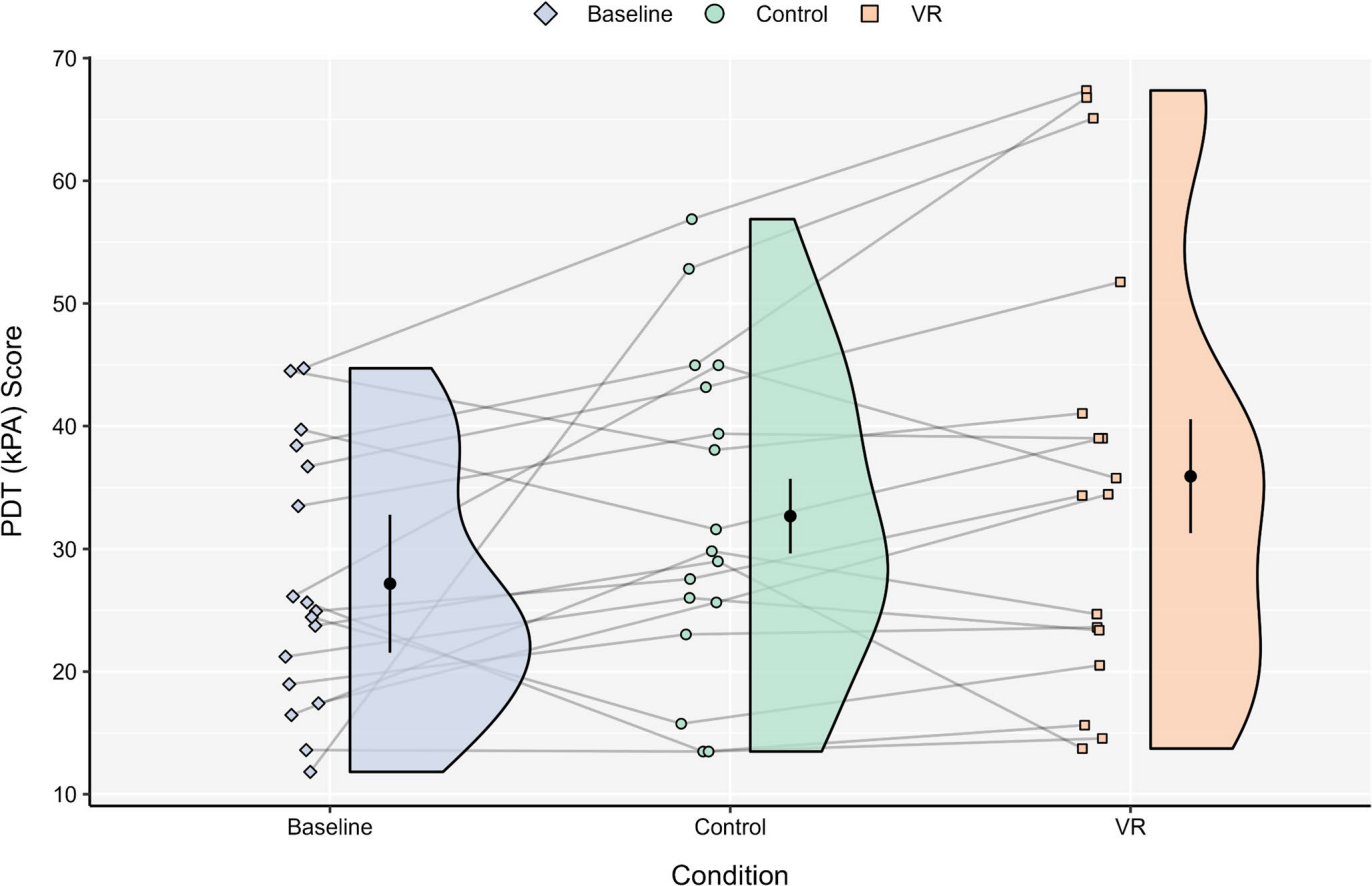
Pressure detection threshold responses at baseline and post-trials. Each density plot is accompanied by the mean and 95% CI. PDT = pressure detection threshold; VR = virtual reality.

A one-way RM ANOVA on PDT scores indicated a significant main effect of assessment (*p* = .038, η_p_^2^ = 0.21). The baseline scores were lower than both the control (*M*_diff_ = − 5.51) and VR (*M*_diff_ = − 8.75) conditions (see Fig. 3), albeit that the Holm–Bonferroni adjustment rendered these comparisons non-significant (*p*s > .05). A one-way RM ANOVA indicated non-significant main effects of assessment for conditioned PDT (*p* = .693, η_p_^2^ = 0.02). We conducted an exploratory (i.e., non-registered) analysis of the ∆PDT (CPM effect) scores across conditions (i.e., baseline, control, VR). A one-way RM ANOVA indicated a non-significant main effect of assessment (*p* = .216, η_p_^2^ = 0.09; see Table 4).

**Table 4:** Inferential statistics for dependent variables

	F	df	*p*	η_*p*_^2^
Pain variables				
PDT				
Assessment	4.24	1, 23	0.038	0.21
PDT (conditioned)				
Assessment	0.37	2, 32	0.693	0.02
∆PDT (CPM effect)				
Assessment	1.61	2, 32	0.216	0.09
Affective variables				
Affective valence				
Condition × timepoint	0.24	3, 48	0.869	0.02
Condition	13.07	1, 16	0.002	0.45
Timepoint	4.70	3, 48	0.006	0.23
Arousal				
Condition × timepoint	0.64	3, 48	0.592	0.04
Condition	3.79	1, 16	0.069	0.19
Timepoint	3.41	2, 28	0.053	0.18
Perceptual variables				
Perceived exertion				
Condition × timepoint	0.72	3, 48	0.545	0.04
Condition	0.30	1, 16	0.593	0.02
Timepoint	25.99	1, 22	< 0.001	0.62
State attention				
Condition × timepoint	0.07	2, 25	0.888	0.01
Condition	5.21	1, 16	0.036	0.25
Timepoint	3.17	1, 21	0.079	0.17

PDT = pressure detection threshold; CPM = conditioned pain modulation

### Affective responses

**Fig. 4:**
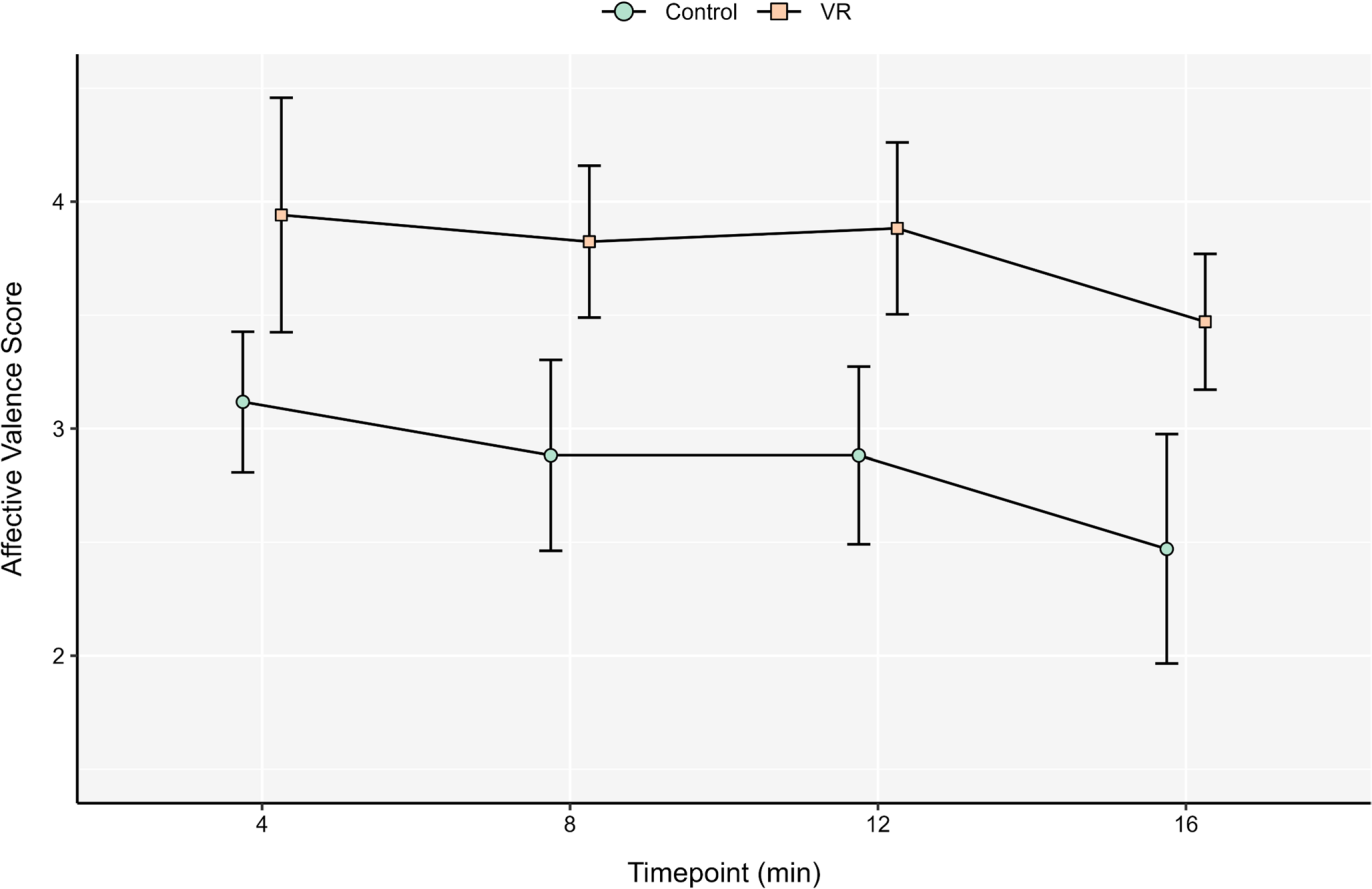
Feeling scale responses between conditions. Error bars denote 95% CIs. Data are dodged on the *x*-axis to prevent over-plotting. VR = virtual reality.

A paired samples *t*-test on affective valence indicated a non-significant effect of condition at minute 0 *t*(16) = − 1.10, *p* = .288 and hence these data were not used as a covariate during subsequent analyses. A factorial RM ANOVA indicated a non-significant 2 (Condition) × 4 (Timepoint) interaction for affective valence during the main exercise phase (*p* = .869, η_p_^2^ = 0.02; see Fig. 4). There was, however, a main effect of Condition (*p* = .002, η_p_^2^ = 0.45), in which the VR condition prompted more positive affective valence when compared to the control condition (*M*_diff_ = 0.94; see Table 4). There was also a main effect of Time (*p* = .006, η_p_^2^ = 0.23), with affective valence scores steadily declining throughout the exercise bout. A factorial RM ANOVA indicated a non-significant 2 (Condition) × 4 (Timepoint) interaction for affective arousal during the main exercise phase (*p* = .592, η_p_^2^ = 0.04), as well as non-significant main effects (*p*s > .05; see Table 4).

### Perceptual responses

A factorial RM ANOVA indicated a non-significant 2 (Condition) × 4 (Timepoint) interaction for RPE (*p* = .545, η_p_^2^ = 0.04). However, there was a main effect of Time (*p* < .001, η_p_^2^ = 0.62), with RPE steadily increasing throughout the exercise bout. The state attention analysis was non-significant in relation to a 2 (Condition) × 4 (Timepoint) interaction effect (*p* = .888, η_p_^2^ = 0.01). Nonetheless, there was a significant main effect of Condition (*p* = .036, η_p_^2^ = 0.25), with the VR condition prompting greater attentional dissociation when compared to control (*M*_diff_ = 16.69).

### Post-task psychological responses

**Fig. 5:**
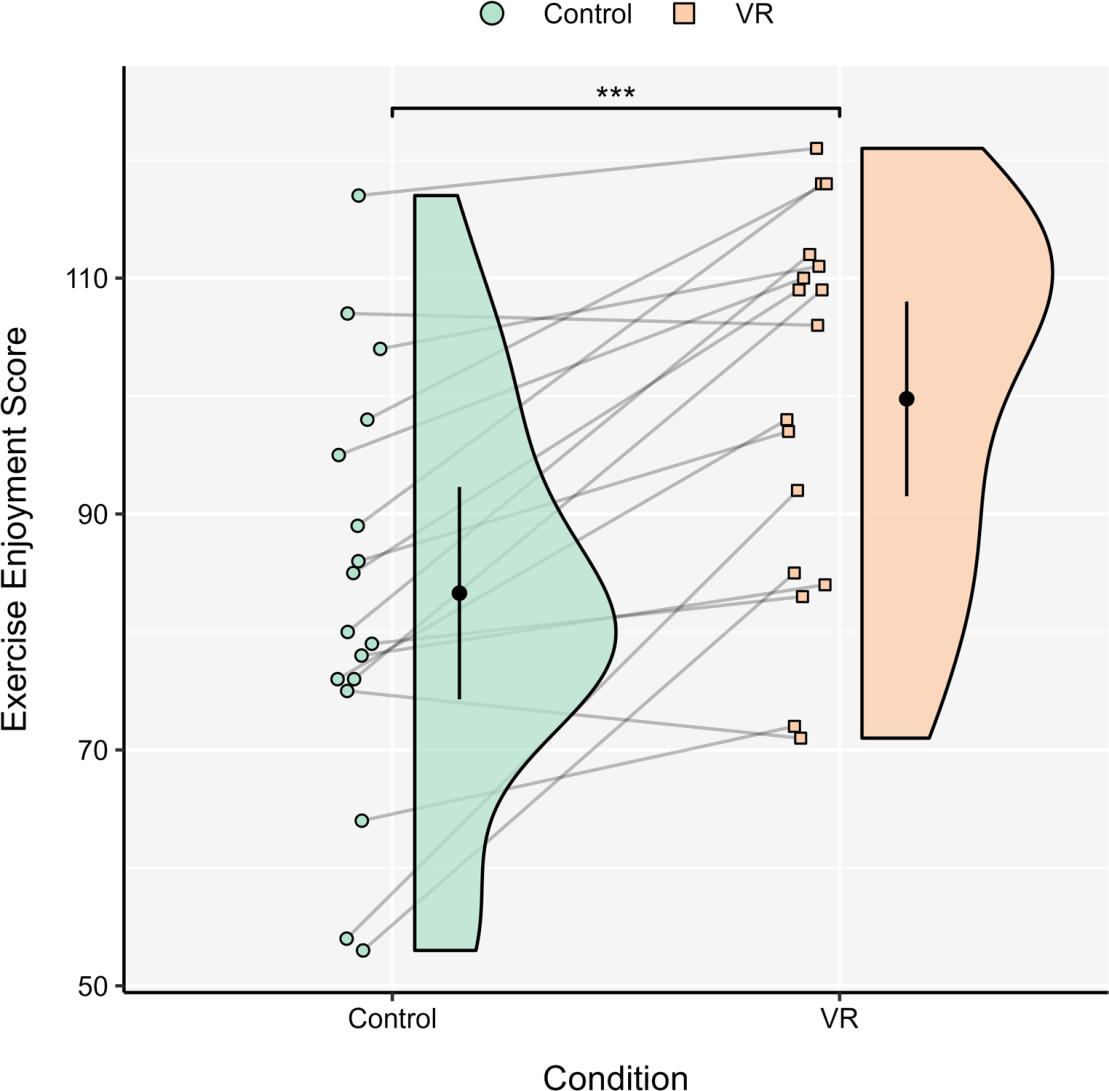
Exercise enjoyment scores between conditions. Each density plot is accompanied by the mean and 95% CI. VR = virtual reality. ***p < .001

Paired samples *t*-tests were significant for exercise enjoyment (*t*(16) = − 5.12, *p* < .001; see Fig. 5), remembered pleasure (*t*(16) = − 2.18, *p* = .045), and forecasted pleasure (*t*(16) = − 2.34, *p* = .033). On all occasions, the VR condition prompted higher scores when compared to control. The VR application was also deemed to prompt high perceptions of presence (*M* = 29.94, *SD* = 5.08) according to the SPES (Hartmann et al. 2016).

## Discussion

The purpose of the study was to examine the effects of VR on experimentally-induced pain (using cuff pressure algometry), as well as affective and perceptual responses to moderate-intensity exercise performed at VT. The results led to acceptance and non-acceptance of the hypotheses, each of which are considered herein. *H*_1−2_ held that the VR condition would enhance exercise-induced effects on PDT and conditioned PDT, respectively. These hypotheses were not accepted, given that the associated post-hoc comparisons between VR and control were non-significant. *H*_3−6_ predicted that the VR condition would prompt greater affective valence, higher affective arousal, reduced RPE, and greater attentional dissociation, respectively, when compared to control. We found main effects of condition for affective valence and state attention (see Table 4), but not affective arousal or RPE. Accordingly, *H*_3_ and *H*_6_ are accepted but *H*_4_ and *H*_5_ are not. Finally, *H*_7_ held that the VR condition would elicit higher perceived enjoyment, remembered pleasure, and forecasted pleasure when compared to control. This hypothesis is accepted on the basis of significant paired samples *t*-tests.

### Pain responses

The results indicated a main effect of assessment for PDT, but not for conditioned PDT responses or the overall CPM effect. These data suggest exercise or exercise with VR can influence pain thresholds but appear to have no effect when endogenous pain modulatory systems are activated. The Holm-Bonferroni adjusted post-hoc comparisons were statistically non-significant and it is plausible that this could be attributed to insufficient statistical power — a limitation of the study.

Several mechanisms have been proposed to explain exercise induced analgesia, most notably the activation of top-down endogenous analgesic systems linked to both opioidergic and serotonergic mechanisms as well as immune activation (Sluka et al. 2018). However, exercise did not appear to affect CPM, which might indicate that the specific mechanisms underlying CPM and exercise-induced analgesia are not identical. CPM refers to pain inhibition or facilitation by the presence of a diffuse painful or noxious stimulus, and it is thought to involve spinally projecting noradrenergic mechanisms (Kucharczyk et al. 2023). While both exercise-induced analgesia and CPM are forms of endogenous pain modulation, they are likely to be mediated by different analgesic circuits (Bannister and Hughes 2023). This distinction between central pain modulatory pathways is crucial for understanding why exercise may affect PDT but not CPM. It is also recognised that CPM responses are inherently variable, with healthy pain-free populations showing several responses ranging from inhibitors, facilitators, and non-responders (Crawford et al. 2025). Therefore, it is possible that these variable responses could be driving the lack of effect seen at the group level for CPM. Future studies should aim to run larger CPM stratified experiments to see whether exercise preferentially influences certain inhibitory or faciliatory profiles.

It was anticipated that VR would enhance the hypoalgesic effects of exercise by further activating attention-based pain modulation mechanisms. Previous research has shown that VR can reduce pain perception by engaging an individual’s attention in an immersive environment, which diverts focus away from pain, reducing its perceived intensity (Hoffman et al. 2000; Li et al. 2011; Hughes et al. 2019; Trost et al. 2021). We anticipated that VR would amplify the analgesic effects of exercise by facilitating attentional dissociation away from interoceptive cues, thereby improving affective responses. However, the results of this study were somewhat unexpected in that the VR condition improved affective and perceptual responses, but did not significantly enhance the analgesic effects of exercise on PDT.

It is possible that the analgesic mechanisms activated during exercise might already be sufficient to provide a significant pain-relieving effect on PDT. EIH is a well-established phenomenon that activates robust pain-modulating pathways, and VR may not be able to further enhance these inhibitory effects if the underlying neural circuits are already maximally engaged (Koltyn 2000). Additionally, participants in the VR condition reported greater attentional dissociation, which likely contributed to the increased enjoyment and positive affective responses. However, this attentional shift may not have been sufficient to alter the underlying pain-modulatory processes activated by exercise (Vogel et al. 2022). The effects of VR on endogenous pain modulatory systems are likely more complex and may depend on factors such as the type of VR environment, the intensity of the exercise, and the duration of the VR exposure (Tesarz et al. 2024). It is noteworthy that the VR condition prompted high scores for presence (see Table 3), indicating that participants strongly felt being transported to the virtual countryside scene during exercise (Slater 2018).

### Affective responses

Despite the lack of enhancement in experimentally-induced pain modulation, VR prompted greater affective valence during the exercise bout when compared to control. This was associated with a large effect size (see Table 4) that broadly aligns with findings from the extant literature (Bird et al. 2024). A key tenant of psychological hedonism is that individuals are likely to repeat behaviours that result in pleasure and avoid behaviours that result in displeasure (Kahneman 1999). Such feeling states have been shown to be indicative of future exercise behaviour. For example, Teixeira et al. (2024) reported that an instructional intervention designed to enhance affective valence facilitated a 77% increase in attendance at an exercise facility over an 8-week period, when compared to control. Accordingly, VR technology could hold practical utility in broadening the appeal of exercise, particularly among the 1.8b adults who are insufficiently active globally (Strain et al. 2024).

The VR condition elicited higher affective arousal scores during the exercise bout when compared to control, albeit that this did not reach statistical significance. This finding was somewhat unexpected and stands in opposition to previous investigations involving the use of immersive technology in an exercise context (Bird et al. 2021). It has been suggested that novel stimuli prompts higher arousal when compared to familiar stimuli (Weierich et al. 2010). Hence, it is plausible that the lack of differences in arousal between conditions observed here could be indicative that VR is beginning to shed its novelty as this form of technology matures.

### Perceptual responses

The VR condition prompted lower RPE scores throughout the exercise bout when compared to control. However, this did not reach statistical significance. The effects of VR technology on exercise-related RPE have been largely inconclusive to date (Matsangidou et al. 2019; Bird et al. 2024). Bruce et al. (2025) found that cycling on a virtual hill with a 9% gradient prompted higher RPE when compared to cycling on a virtual hill with a 6% gradient, despite a constant workload. Hence, there appears to be potential in modifying RPE when VR content is incongruent to the associated exercise workload, albeit that we did not seek to incorporate this feature in the present experimental manipulation. We observed a main effect of time for RPE, in alignment with related investigations (Bird et al. 2024), but this was expected to occur over the course of a 15-min exercise bout at VT.

Regarding state attention, the VR condition was effective in eliciting a more dissociative focus when compared to the control condition, in alignment with the extant literature (Moore and Butler 2024). Distraction is a frequently cited technique to encourage behaviour change (Knittle et al. 2020). In an exercise context, attentional dissociation helps individuals divert their focus away from interoceptive cues (e.g., elevated heart rate), making the exercise experience more pleasurable (Bird et al. 2025). VR technology has been shown to prompt greater dissociation during exercise when compared to a range of related technologies, such as augmented reality and television (Bird et al. 2024), making it an appealing technology for exercise-related interventions. The fact that we found a main effect of condition for state attention but not for RPE is in alignment with previous investigations into the uses of music (Feiss et al. 2021) and VR (Bird et al. 2021) during exercise. Collectively, these findings suggest that different neural circuits are implicated when these constructs are subjected to self-assessment; but additional research is required to examine this more fully from a neurophysiological perspective (Bird et al. 2025).

### Post-task psychological responses

The VR condition engendered greater post-task scores for enjoyment, remembered pleasure and forecasted pleasure, when compared to control. These findings contribute towards a growing corpus of research demonstrating the positive application of VR technology in an exercise context (Bird et al. in press; Barbour et al. 2024). The positive responses reported by participants in the VR condition highlight the potential of the technology to improve the affective experience of exercise. This may, in turn, lead to better long-term adherence to physical activity regimens, in accord with psychological hedonism (Kahneman 1999).

### Strengths and limitations

A core strength of the study was the use of cuff pressure algometry to examine the influence of VR technology on exercise-related pain. Cuff pressure algometry is a robust, semi-automated and reliable approach used to assess pain processing (Graven-Nielsen et al. 2015). Another strength was that the exercise intensity employed during experimental trials was informed by the Dual-Mode Theory (Ekkekakis 2003). This holds that there is great inter-individual variability in affective valence at the VT, making it an appropriate intensity for the use of audio-visual interventions such as VR. Relatedly, each participant was required to complete an incremental exercise test prior to experimental trials. This allowed us to ascertain the workload that corresponded with each participant’s VT and minimise between-subject variability. Finally, the investigation was predicated on a range of Open Science practices (e.g., registration, data availability) as a means of improving research integrity (Hagger 2022).

A limitation of the study is the relatively small sample size, as well as the inconsistencies encountered in relation to the PPT and TSP protocols. Another limitation concerns responding to self-assessment inventories while wearing a VR headset (Bird et al. 2021). However, we sought to reduce the severity of this by only administering single-item measures during the exercise bout. Furthermore, each participant was fully habituated to the measures prior to experimental trials and the order of implementation was held constant throughout (i.e., FS, FAS, CR10, AS). Another limitation is that previous and current pain experiences were not accounted for in our eligibility criteria. Indeed, the sample comprised young, healthy, mostly British adults (see Table 1) who frequently engaged in physical activity. Accordingly, the findings of the study should not be generalised prior to replication with other subgroups of the population (e.g., inactive adults, chronic pain patients).

### Implications for practice and future research

A clear implication to emerge from the present investigation is that immersive VR technology can significantly enhance the affective experience of exercise. This was evidenced by both in-task ratings of affective valence and post-task ratings of exercise enjoyment, remembered pleasure, and forecasted pleasure. These findings are apposite in light of the “rise of affectivism” (Dukes et al. 2021, p. 816), in which researchers are increasingly recognising that affective responses are powerful motivators of human behaviour. Another implication is that VR technology offers a potent means of promoting attentional dissociation during exercise, which could be of particular value among those developing behaviour change interventions (Knittle et al. 2020). A further advantage of VR is the degree of interactivity afforded by the technology when compared to traditional means of displays, such as televisions (Bird et al. 2024). This means that specific exercises can be encouraged by practitioners and tracked in real-time through advanced sensors embedded in VR-related hardware.

There are several avenues for future research. From a practical perspective, investigators might seek to replicate the study with patient populations to optimise VR as a tool for improving exercise engagement and pain management. From a methodological perspective, there is scope to explore the effects of different virtual environments beyond countryside scenes, such as those traditionally encountered during blue exercise (Thompson and Wilkie 2021). Scholars might also examine how VR technology might assist in lowering RPE during exercise through the use of incongruent visuals (see e.g., Bruce et al. 2025) or subtle manipulations to virtual avatars (see e.g., Kocur et al. 2022). From a mechanistic perspective, there are opportunities to examine neurophysiological responses to VR exercise using technologies such as electroencephalography (EEG) and functional near-infrared spectroscopy (*f*NIRS) to gain a deeper understanding of the top-down circuitry involved (Medina et al. 2024).

### Conclusions

The current findings indicate that while VR technology did not amplify the analgesic effects of exercise, it did positively influence the affective experience, as well as prompting greater attentional dissociation. The improvement of affective valence and attentional dissociation during exercise could play a crucial role in increasing exercise adherence, particularly among physically inactive individuals. Increased adherence to exercise programmes would feasibly lead to more favourable long-term outcomes for physical and mental health. Accordingly, VR technology might be considered by practitioners as a useful tool through which to facilitate pleasurable exercise experiences.

## Data Availability

Data and code are available via the Open Science Framework (https://doi.org/10.17605/OSF.IO/BU85T).
